# Second generation PSMA-targeted turn-on probe for imaging cargo release in prostate cancer cells

**DOI:** 10.1016/j.bmcl.2025.130530

**Published:** 2025-12-31

**Authors:** Nooshin Mesbahi, Brenna C. McAllister, Hosog Yoon, Aaron T. Hendricksen, Melody D. Fulton, Leslie A. Caromile, Clifford E. Berkman

**Affiliations:** a Washington State University, Department of Chemistry, Pullman, WA 99164–4630., United States; b University of Connecticut, Center for Vascular Biology, Farmington, CT 06030-3501, United States

**Keywords:** PSMA, turn-on probe, phosphoramidate, pH-responsive cleavable linker

## Abstract

Targeted payload release in cancer cells can be modulated by tuning both the linker, spacer, and the payload chemistries. In previous studies, a PSMA-targeted probe incorporating a 7-amino-4-methylcoumarin (AMC) payload and a PEG linker resulted in predominant payload release in the lysosome (pH ~5.0). Here, we introduce a second-generation PSMA-targeted turn-on probe with a shorter, hydrophobic linker and a 7-hydroxy-4-methylcoumarin (HMC) payload. Based on pH-dependent kinetic studies, the HMC payload exhibits faster cleavage at a slightly higher pH (pH 5.5), suggesting an earlier release—potentially more in early endosomes than lysosomes. Our results demonstrate that subtle changes in linker and payload structures can alter intracellular release kinetics, offering improved control over the cellular release site, which is critical for optimizing targeted therapeutic and imaging strategies in prostate cancer cells.

Prostate cancer cells overexpress prostate-specific membrane antigen (PSMA), a type II transmembrane protein, and its expression levels are strongly correlated with the course of the disease, castration resistance, and potential for metastasis.^1–4^ Due to its strong receptor-mediated internalization and limited expression in healthy tissues, PSMA is an ideal biomarker and therapeutic target. Many PSMA-targeted therapeutic and diagnostic agents have been developed as a result of this distinct profile, including imaging probes, antibody-drug conjugates (ADCs), and regulatory-approved radiotherapeutics, such as 68Ga-PSMA-11 and piflufolastat F-18.^5–23^ Despite their potential, ADCs have limitations, including immunogenicity, off-target toxicity, and inadequate internalization.^24–26^ To enhance tumor selectivity and intracellular delivery, small-molecule drug conjugates (SMDCs) have been explored, which combine cytotoxic payloads, cleavable linkers, and high-affinity PSMA ligands.

With PSMA-targeted drug delivery, precise control over intracellular payload release is crucial for achieving therapeutic efficacy and safety. PSMA-targeted SMDCs utilize receptor-mediated endocytosis to selectively internalize agents into cancer cells. The release of the payload is controlled by subcellular conditions, such as pH or enzymatic activity.^27^ Recent developments in our lab have shown that it may be possible to control whether the payload is released under the pH conditions of lysosomes or early endosomes by tuning the linking chemistry to the payload.^28–30^ Lysosomal localization was predominantly demonstrated in our earlier work using an aniline-based payload; however, initial pH-dependent kinetics data suggested that a greater amount of a phenolic-type payload could be released under slightly higher pH conditions, potentially shifting its release to early endosomes.^29^

In this study, we report the modular synthesis and evaluation of a second-generation PSMA-targeted turn-on probe (PSMA-TOP2) incorporating a shorter, hydrophobic spacer with a phenolic-type 7-hydroxy-4-methylcoumarin (HMC) payload linked through a pH-responsive linker (Figure 1). We have previously reported on the use of this pH-responsive phosphoramidate linker in targeted delivery applications both *in vivo* and *in vitro*.^31–33^ Based on our preliminary studies comparing the pH-dependent release kinetics of both 7-amino-4-methylcoumarin (AMC) and HMC from the linker, it was expected that more HMC would not be released in the lysosomes, in contrast to what we observed for the AMC payload.^29^ Thus, this study aimed to determine whether subtle pH sensitivity of the linker can influence the subcellular spatiotemporal release of biomarker-targeted payloads.

Guided by these design principles, we synthesized the HMC-based PSMA-targeted turn-on probe (PSMA-TOP2) using a three-step modular conjugation strategy (Scheme 1). In this construct, the PSMA-targeting moiety (DBCO-C6-1298) was coupled to the HMC payload via our established pH-responsive PhosAm linker.^28^ Compared to the first-generation PSMA-TOP,^29^ PSMA-TOP2 incorporates a shorter adipoyl spacer, a modification intended to maintain high-affinity receptor binding while improving atom economy and compactness of the final conjugate. Briefly, Fmoc-protected phosphoramidate ester (**1**), available from prior studies^29,30^ was deprotected with DBU and reacted with bis (4-methyl-2-oxo-2H-chromen-7-yl) carbonate (**2**) to afford a silyl-protected precursor (**3)**. After global deprotection with cesium fluoride to yield compound (**4)**, strain-promoted click-chemistry with DCBC-C6-1298^31^ provided the final PSMA-TOP2.

Based on our previous work, which detailed the pH-dependent hydrolysis kinetics of coumarin-based payloads via our pH-responsive PhosAm linker, payload release was generally negligible at physiological pH, regardless of the payload. However, we observed that the phenolic-based payload HMC underwent maximal release at pH 5.5, whereas the aniline-based AMC was released maximally at pH 5.0. Those results suggested that greater payload release from the PhosAm linker would consequently be observed in the higher pH of endosomes rather than in lysosomes, based on the nature of the linker.^29^

To assert and investigate the intracellular trafficking and pH-dependent release behavior of PSMA-TOP2, we performed quantitative fluorescence colocalization studies in PSMA-positive (C42B-PSMA^Scramble^) and PSMA-negative (C42B-PSMA^Knockout^) human prostate cancer cells.^29^ In our experiments, coumarin fluorescence was used as a surrogate for the release of intracellular payloads, enabling the spatial mapping of release dynamics. We chose to quantify the colocalization signal of coumarin with cellular organelles, such as the lysosome and the early endosome in two ways. First, we used the JACoP plugin in FIJI/ImageJ across ≥10 fields per condition, with Pearson's correlation coefficients (r) used to assess signal overlap. Based on a published correlation scale, a correlation of <0.20 is very weak, a correlation between 0.20–0.39 is weak, a correlation between 0.40–0.59 is moderate, a correlation between 0.60–0.79 is strong, and a correlation>0.80 is very strong.^34^ However, the Pearson correlation coefficient cut-offs are set arbitrarily to refer to linear associations, which do not always exist.^34^ Therefore, to further validate our data and obtain a more accurate measurement of co-localization throughout the entire section, we used FIJI/ImageJ to calculate the area of coumarin co-localization as a percentage of the intracellular compartment of interest.

To first confirm the specificity of PSMA-TOP2 for cell-surface PSMA and to ensure the selective release of its fluorogenic payload within PSMA-positive cells only, we incubated 10 μM of the conjugate with our PSMA-positive and PSMA-negative prostate cancer cells for 30 minutes at 37°C. FIJI/ImageJ fluorescence colocalization analysis revealed that 21% of the released HMC colocalized with intracellular PSMA (visualized with PSMA antibody Huj591-Gsmab) exclusively in C42B-PSMA^Scramble^ cells (Figure 2). Additionally, FIJI JACoP revealed a Pearson's Correlation Coefficient of r = 0.784 (Figure S1, Supplemental Information), indicating a moderate to strong linear association. In contrast, no significant colocalization of PSMA with the fluorescent HMC signal was detected on the cell surface or within C42B-PSMA ^Knockout^ cells (Figure 2). Taken together, the data indicate that PSMA-TOP2 binds to cell-surface PSMA exclusively, undergoes endocytosis, and releases its fluorogenic payload within intracellular compartments.

PSMA-TOP2 was expected to exhibit a higher pH activation threshold than our previously published AMC-bearing PSMA-conjugate.^29^ Thus, earlier release within the endolysosomal continuum was anticipated. However, following 30-minute incubation with 10 μM PSMA-TOP2, FIJI/ImageJ fluorescence colocalization analysis and FIJI JACoP analysis revealed that 17% (Pearson's r = 0.667) of the coumarin-derived fluorescent signal colocalized within the pH 4.5–5.5,^29,35^ LAMP1-positive lysosomal compartment (Figure 3 and Figure S1 Supplemental Information), and 12% localized (Pearson's r = 0.651) to the higher pH EEA1-positive early endosomal compartment (pH 5.9–6.8) (Figure 4 and Figure S1, Supplemental Information).^35^ By contrast, the AMC-conjugated probe previously demonstrated >98% lysosomal colocalization with minimal early endosomal release.^29^ Notably, approximately 67% of the HMC-derived signal was diffusely distributed throughout the cytosol, with no significant colocalization with LAMP1 or EEA1. This signal was confined within the cell membrane boundaries, indicating intracellular release rather than extracellular leakage. However, the observed extent of premature release, particularly the significant signal within early endosomes and the diffuse cytosolic distribution, was greater than expected, suggesting that PSMA-TOP2 may be inherently less stable under physiological conditions.

Lysosomal trafficking plays a critical role in determining the intracellular fate of endocytosed therapeutics.^37^ Targeted agents, such as antibody-drug conjugates and pH-sensitive nanoparticles, are often engineered to exploit lysosomal acidity to trigger intracellular drug release.^36,37^ In the case of PSMA-targeted therapies, ligand-receptor internalization directs the therapeutic conjugate through the endosomal pathway, culminating in the lysosome, a compartment enriched in acidic hydrolases that are ideal for activating cleavable linkers or releasing cytotoxic payload.^38^ This lysosomal routing is not only essential for drug activation but also for preventing premature degradation or trapping in non-productive intracellular compartments.^39^ Moreover, tumor types with elevated lysosomal biogenesis, such as castration-resistant or neuroendocrine prostate cancer, may be particularly amenable to lysosome-targeted strategies,^40^ thereby offering an opportunity to exploit tumor-specific vulnerabilities.

The premature appearance of the signal in early endosomes and widespread cytosolic dispersion deviates from the intended lysosome-specific release profile. Given that the early endosomal pH (5.9–6.8) closely mimics the acidic tumor microenvironment,^41^ this early release raises concerns about nonspecific extracellular activation and potential bystander effects. Furthermore, lysosomal confinement is important for minimizing off-target toxicity and enabling selective activation of cleavable linkers by resident hydrolases. Compared to our previous AMC probe, PSMA-TOP2 exhibited less lysosomal release due to subtle chemical differences affecting pH-responsiveness and intracellular routing.

In summary, this study presents the preparation and use of a second-generation PSMA-targeted turn-on probe as a surrogate for a PSMA-targeted drug-conjugate to report on the spatiotemporal payload release in PSMA(+) cells. PSMA-TOP2 differs from our first-generation probe^29^ in that it incorporates a short hydrophobic spacer,^31^ the phenolic HMC payload, while maintaining the small-molecule PSMA ligand (CTT1298) and a pH-responsive PhosAm linker.^28,29^ Owing to the more rapid release kinetics of the HMC payload at pH 5.5 than at pH 5.0, it was expected that greater HMC release (turn-on fluorescence) would not be observed in lysosomes, which was consistent with the observations reported herein (Figure 3). This study, together with our previous study,^29^ underscores the influence that subtle payload modifications can have on drug activation in applications focused on biomarker-targeted conjugates and that there exists the potential for fine-tuning therapeutic efficacy by exploiting distinct pH-sensitivities of the payload linkages. Furthermore, these findings highlight the necessity of empirically validating pH-sensitive linker designs in cells: even modest changes in payload chemistry can shift subcellular trafficking and compromise compartmental confinement. For PSMA-targeted delivery applications, early cytosolic release raises concerns about nonspecific activation, bystander effects, and off-target toxicity. Future linker–payload strategies must therefore balance biochemical tunability with payload properties, particularly lipophilicity and charge, to ensure precise spatial and temporal control of drug activation in PSMA-expressing tumors.

## Supplementary Material

1

## Figures and Tables

**Fig. 1.**
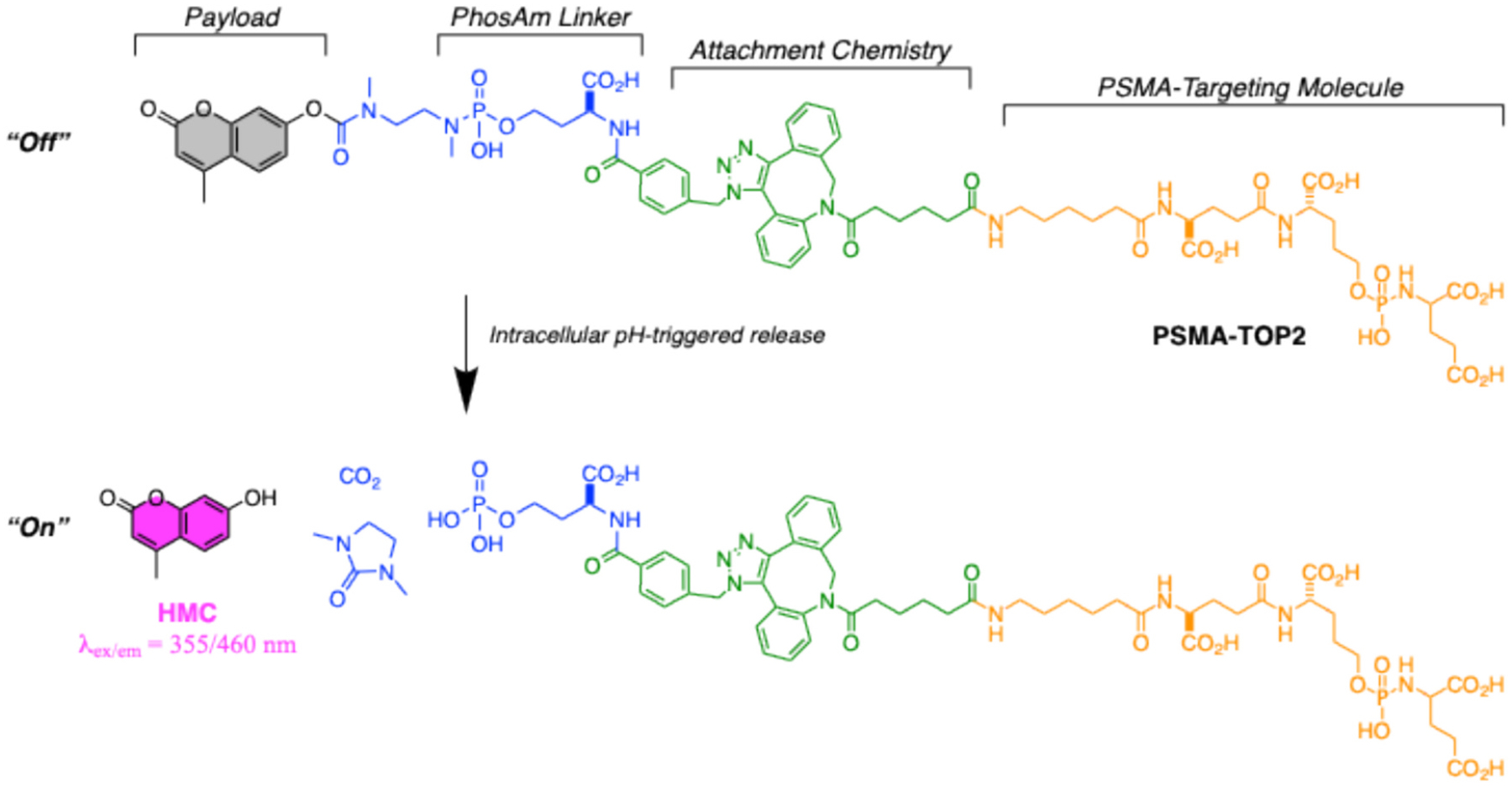
Design and functional action of the PSMA-targeted turn-on probe PSMA-TOP2.

**Fig. 2.**
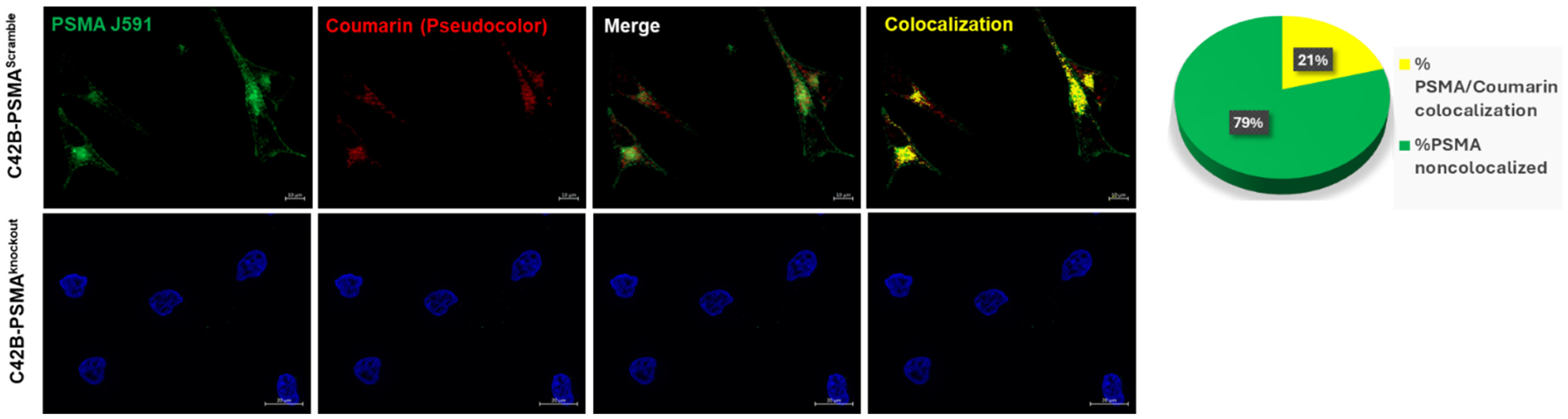
PSMA-TOP2 binds cell surface PMSA and is internalized via receptor-mediated endocytosis. Immunofluorescent microscopy imaging of C42B-PSMA scramble and C42B-PSMA^knockout^ cells incubated with a PSMA-TOP2 (pseudocolor red) and PSMA antibody J591 (green). FIJI/ImageJ fluorescence colocalization analysis indicates the percentage of colocalization between PSMA and the released coumarin (yellow). 63× oil, DAPI blue.

**Fig. 3.**
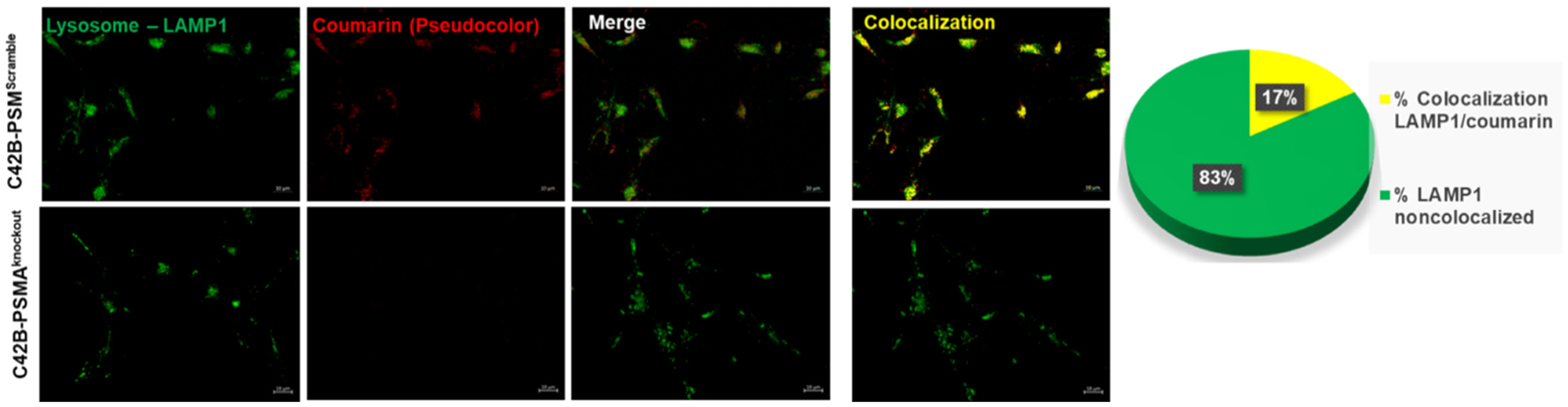
A PSMA-TOP2 releases its payload in the low pH environment of the lysosome. Immunofluorescent microscopy imaging of C42B-PSMA scramble and C42B-PSMA ^knockout^ cells incubated with a PSMA-TOP2 (pseudocolor red) and LAMP1 antibody (green). FIJI/ImageJ fluorescence colocalization analysis indicates the percentage of colocalization between LAMP1 and the released coumarin (yellow). 63× oil.

**Fig. 4.**
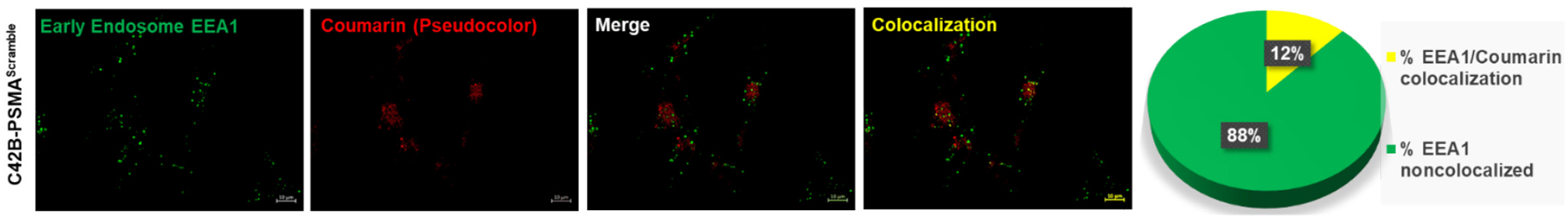
A PSMA-TOP2 releases its payload in the early endosome. Immunofluorescent microscopy imaging of C42B-PSMA ^scramble^ and C42B-PSMA ^knockout^ cells incubated with a PSMA-TOP2 (pseudocolor red) and EEA1 antibody (green). FIJI/ImageJ fluorescence colocalization analysis indicates the percentage of colocalization between EEA1 and the released coumarin (yellow). 63× oil.

**Scheme 1.**
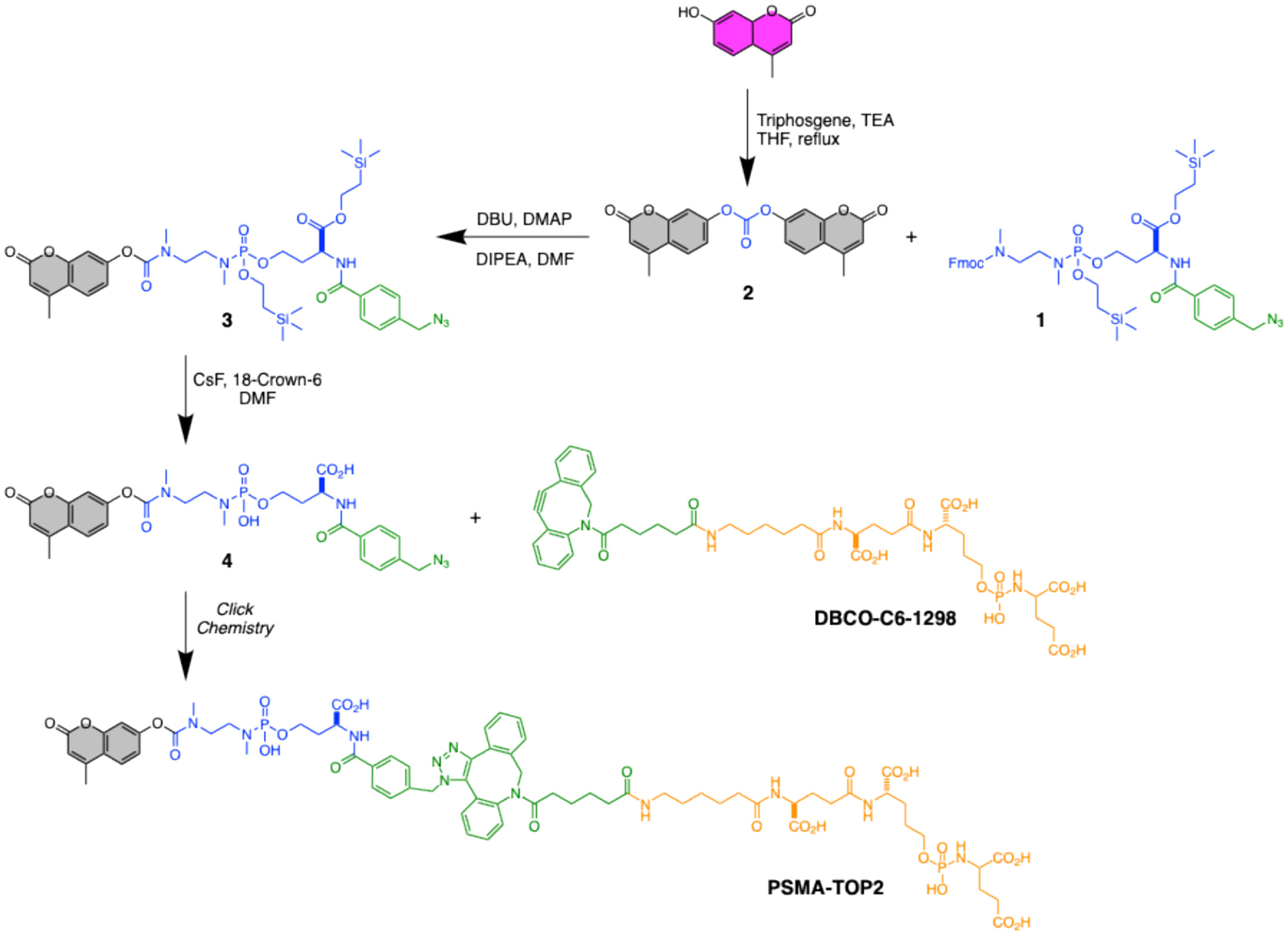
Modular Assembly of the pH-responsive PSMA turn-on probe **PSMA-TOP2**.

## Data Availability

Data will be made available on request.

## Appendix A. Supplementary data

For detailed synthetic procedures, characterization data, and other experimental details, please refer to the Supplemental Material. Supplementary data to this article can be found online at [https://doi.org/10.1016/j.bmcl.2025.130530.
