# Supplementary material for: Second generation PSMA-targeted turn-on probe for imaging cargo release in prostate cancer cells
Source: Bioorg Med Chem Lett. Author manuscript; Available in PMC 2026 Jul 1. (PMC13322305; doi:10.1016/j.bmcl.2025.130530)
Supplement: 1 [file NIHMS2189209-supplement-1.docx]

**SUPPORTING INFORMATION**

**Second Generation PSMA-Targeted Turn-on Probe for Imaging Cargo Release in Prostate Cancer Cells**

Nooshin Mesbahi ^a^, Brenna C. McAllister ^b^, Hosog Yoon ^a^, Aaron T. Hendricksen ^a^,

Melody D. Fulton ^a^, Leslie A. Caromile ^b^, and Clifford E. Berkman*^a^

Washington State University, Department of Chemistry, Pullman, WA 99164–4630.

University of Connecticut, Center for Vascular Biology, Farmington, CT 06030-3501.

*Corresponding author. Tel.: +1-509-335-1923; e-mail: [cberkman@wsu.edu](mailto:author@university.edu)

**Table of Contents**

1. PSMA-targeted turn-on probe (PSMA-TOP2) Synthesis
2. Cell Surface Labeling and Internalization Studies
3. Supplementary Figure 1
4. NMR Spectra of PSMA-TOP2 and Key Intermediates
5. HRMS Spectrum of PSMA-TOP2
6. HPLC Methods and Chromatogram
7. Inhibitory Potency (IC_50_) of TOP2 to PSMA

**I. PSMA-targeted turn-on probe (PSMA-TOP2) Synthesis**

All solvents employed in the reactions were anhydrous, sourced commercially or freshly distilled over calcium hydride prior to use. Nuclear Magnetic Resonance (NMR) spectra, including **^1^H,** **^13^C**, and **^31^P**, were acquired using Varian 600 MHz or Bruker Avance NEO 500 MHz spectrometers. High-resolution mass spectrometry (HRMS) analyses were performed utilizing an Applied Biosystems 4800 MALDI-TOF/TOF mass spectrometer (Applied Biosystems, Foster City, CA)

**Compound** **1** was prepared from a published procedure.^1^

**Bis(4-methyl-2-oxo-2H-chromen-7-yl) carbonate (2).**

To a 2-neck round-bottom flask (250 mL) under an argon atmosphere was added a solution of 7-hodroxy-4-methylcoumarin (2.47 g, 14.153 mmol) and triphosgene (0.70 g, 2.359 mmol) in anhydrous THF (38 mL). The mixture was heated to 60 °C and allowed to reflux. Triethylamine (1.4 mL) was added dropwise, and the solution was stirred while monitoring the reaction progress via TLC every 30 minutes. Upon formation of a white precipitate of bis(coumarin) carbonate, the mixture was filtered, and the solid was washed with MeOH. The product was dried under vacuum for 1 hour and used directly in subsequent reactions without further purification.

**TLC:** Rf = 0.2 (60% EtOAc in Hexanes, visualization by UV).

**2-(trimethylsilyl)ethyl N-(4-(azidomethyl)benzoyl)-O-((methyl(2-(methyl(((4-methyl-2-oxo-2H-chromen-7-yl)oxy)carbonyl)amino)ethyl)amino)(2-(trimethylsilyl)ethoxy)phosphoryl)-L-homoserinate(3).**

To a stirring solution of **1** (0.5 g, 0.587 mmol) and **2** (0.266 g, 0.704 mmol) in anhydrous DMF (5.7 mL), was added a solution of DMAP (0.961 g, 0.117 mmol), DBU (0.756 mL, 0.117 mmol), and DIPEA (0.988 mL, 0.881 mmol) in anhydrous DMF (5.7 mL) at room temperature under Argon overnight. The reaction solution was diluted with EtOAc (70 mL), washed sequentially with 1N HCl (aq) (50 mL, 3x), sat. NaHCO3 (aq) (50 mL, 2x) and Brine (50 mL, 1x). The organic layer was dried over Na2SO4, EtOAc was removed under reduced pressure and the resulting oil was chromatographed using a silica-gel flash column (50% EtOAc in Hexanes → 65% EtOAc in Hexanes). The isolated product was dried to give a yellow oil.

**TLC:** Rf = 0.26 (60% EtOAc in Hexanes, visualization by UV).

**^1^H NMR (600 MHz, Chloroform-d)** δ 7.85 (ddd, 2H), 7.57–7.45 (m, 2H), 7.29 (dt, 2H), 7.13–6.99 (m, 2H), 6.19–6.13 (m, 1H), 4.82–4.67 (m, 1H), 4.32 (d, 2H), 4.27–4.14 (m, 2H), 4.10–3.92 (m, 4H), 3.62–3.40 (m, 2H), 3.34–3.15 (m, 2H), 3.11–2.96 (m, 3H), 2.71–2.59 (m, 3H), 2.31 (dd, 5H), 0.99 (dt, 4H), –0.06 (s, 18H).

**^31^P NMR (162 MHz, Chloroform-d)** δ 10.72, 10.67, 10.50, 10.42.

**N-(4-(azidomethyl)benzoyl)-O-(hydroxy(methyl(2-(methyl(((4-methyl-2-oxo-2H-chromen-7-yl)oxy)carbonyl)amino)ethyl)amino)phosphoryl)-L-homoserine(4).**

To a stirring solution of CsF (0.147 g, 0.970 mmol) and 18-crown-6 (0.402 mL, 1.552 mmol) in anhydrous DMF (2 mL), was added a solution of **3** (0.322 g, 0.388 mmol) in anhydrous DMF (1.5 mL) dropwisely stirred at ambient temperature under an argon atmosphere. The reaction mixture was stirred for 6 hours at room temperature. Following completion of the reaction, the solvent was removed under reduced pressure, and KHCO₃(0.155 g, 1.55 mmol) was added to the resulting residue. The mixture was dissolved in minimal ddH₂O and chromatographed using a C18 flash column using a gradient solvent elution (10%ACN in H₂O → 60% ACN in H₂O) to give an off-white solid.

**TLC:** Rf = 0.68 (60% MeOH in H₂O, C18, visualized by UV)

**^1^H NMR (600 MHz, Deuterium Oxide)** δ 7.69–7.59 (m, 3H), 7.30 (dd, 2H), 7.10–7.02 (m, 1H), 6.99 (dd, 1H), 6.25 (dd, 1H), 4.45–4.32 (m, 3H), 3.91 (q, 2H), 3.43 (dd, 2H), 3.17–2.93 (m, 5H), 2.60 (dd, 3H), 2.38 (dd, 3H), 2.33–2.22 (m, 1H), 2.05 (dtt, 1H)

**^31^P NMR (162 MHz, Deuterium Oxide)** δ 10.47, 10.13.

**DBCO-C6-1298** was prepared from a published procedure**.** ^2-3^

**PSMA-TOP2.**

Compound **4** (5 mg, 7.55 µmol) was dissolved in 0.5 M KHCO₃/ddH₂O (194 µL) and was added dropwise to a solution of DBCO-C6-1298 (12.35 mg, 11.33 µmol) in the same buffer (129 µL). Reverse-phase TLC revealed completion of reaction within 60 min; however, stirring was prolonged to 3 hours at ambient temperature to secure full consumption of the precursors. Upon completion monitored by reverse-phase TLC, excess **3** was then efficiently scavenged using azide-functionalised agarose resin. The solvents were removed under reduced pressure and the resulting residue was lyophilized overnight. The final desalting on a C18 Sep-Pack cartridge (gradient elution, 100% ddH_2_O → 30% MeOH), affording the desired conjugate as a white solid in 68 % isolated yield.

**TLC**: Rf = 0.62 (60% MeOH in ddH_2_O, visualization by UV).

**^1^H NMR (600 MHz, Deuterium Oxide)** δ 7.59 – 7.53 (m, 7H), 7.23 – 7.19 (dd, 5H), 6.98 – 6.95 (dt, 7H), 6.92 – 6.90 (dd, 7H), 4.33 – 4.25 (m, 7H), 3.84 – 3.76 (ddt, 7H), 3.71 – 3.69 (m, 3H), 3.42 – 3.41 (dd, 1H), 3.37 – 3.32 (dt, 3H), 3.25 – 3.21 (d, 1H), 3.06 – 2.95 (m, 9H), 3.71 – 3.69 (m, 3H), 2.87 (s, 4H), 2.61 (s, 1H), 2.54 – 2.47 (dd, 7H), 2.35 – 2.29 (d, 7H), 2.17 – 2.11 (ddd, 2H), 1.97 – 1.91 (dq, 2H).

**^31^P NMR (162 MHz, Deuterium Oxide)** δ 10.55, 10.20, 6.32, 6.27.

**HRMS (MALDI)**: m/z calculated for C_69_H_85_N_11_O_25_P_2_^-^ [M+H]^-^: 1529.52, found 1528.5115.

**II. Cell Surface Labeling and Internalization Studies.**

**Cells**

The immortalized human prostate cancer cell line C42B-crispr-PSMA^Scramble^ (PSMA-positive) and C42B-Crispr-PSMA^knockout^ (PSMA-negative)^3^ were maintained in RPMI‐1640 medium (Thermo Fisher Pittsburgh, PA), supplemented with 10% fetal bovine serum, 2 mM glutamine, 100 μg/ml pen-strep, and insulin-transferrin-selenium (Thermo Fisher Pittsburgh, PA) in a humid atmosphere containing 5% CO2 at 37°C.

**Antibodies**

The following primary antibodies were used: LAMP1 (D4O1S) Mouse mAb (Cell Signaling, Danvers, MA, Cat#15665) and EEA1 (E9Q6G) Mouse mAb (Cell Signaling, Danvers, MA, Cat# 48453), Huj591-Gsmab Humanized Recombinant Human Monoclonal Antibody (Thermo Fisher, Waltham, MA, Cat# MA5-41736). The following secondary antibodies were used: Goat anti-Mouse IgG (H+L) Cross-Adsorbed Secondary Antibody, Alexa Fluor 488 (Invitrogen, Carlsbad, CA Cat# A11001), Goat anti-Human IgG (H+L) Superclonal Secondary Antibody, Alexa Fluor 488 (Invitrogen, Carlsbad, CA Cat# A66724).

**Software**

Zeiss Zen Blue Software was used to acquire and process immunofluorescent images. Colocalization analysis was done using FIJI colocalization analysis and FIJI JaCOP.

**Statistical Analysis**

All experiments in this study were repeated for a minimum of three independent experiments. Results are presented as mean ± SEM. Statistical analysis was performed using a paired, two-tailed t-test. Differences were considered significant at P < 0·05.

**Immunofluorescence Microscopy**

Cells were plated onto coverslips at a concentration of 1x105 cells/well in 1 ml growth medium and allowed to attach overnight. Cells were then starved for 2 hours in FBS-free RPMI and then incubated for 30 minutes with 10 μM of the hydroxycoumarin PSMA-targeted conjugate at 37°C. For imaging, coverslips were set on ice, rinsed twice in ice-cold PBS, fixed in ice-cold 10% neutral buffered formalin solution for 15 minutes, blocked, and incubated for 60 minutes with either LAMP-1(Cell Signaling, Danvers, MA), EEA-1 (Cell Signaling, Danvers, MA)Huj591-Gsmab (Thermo Fisher) in 1% normal goat serum in humidity chamber 4°C. Slides were washed in PBS and incubated with Alexa Fluor 488 goat anti-mouse (Invitrogen) or Alexa Fluor 488 human anti-rabbit (Invitrogen) for 1 h. Slides were washed in PBS and mounted in VECTASHIELD Hardset Antifade Mounting Medium with or without DAPI (Vector Laboratories.) Images were acquired using a Zeiss LSM510 META based on an Axiovert 200 microscope at 63x oil and processed using the Zeiss Zen Blue software v3.6.


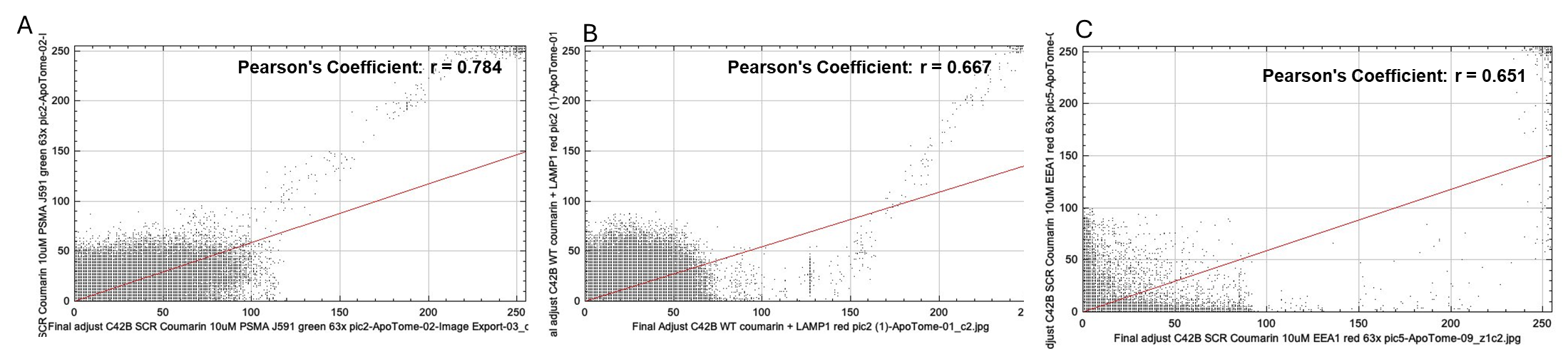


**Supplementary Figure 1: Pearson's coefficient of colocalization determined by FIJI JaCOP**. A. Colocalization of released coumarin and PSMA, B. Colocalization of released coumarin and LAMP1, C. Colocalization of released coumarin and EEA.

**III. Supplementary Figure 1**

**IV. NMR Spectra of PSMA-TOP2 and Key Intermediates**


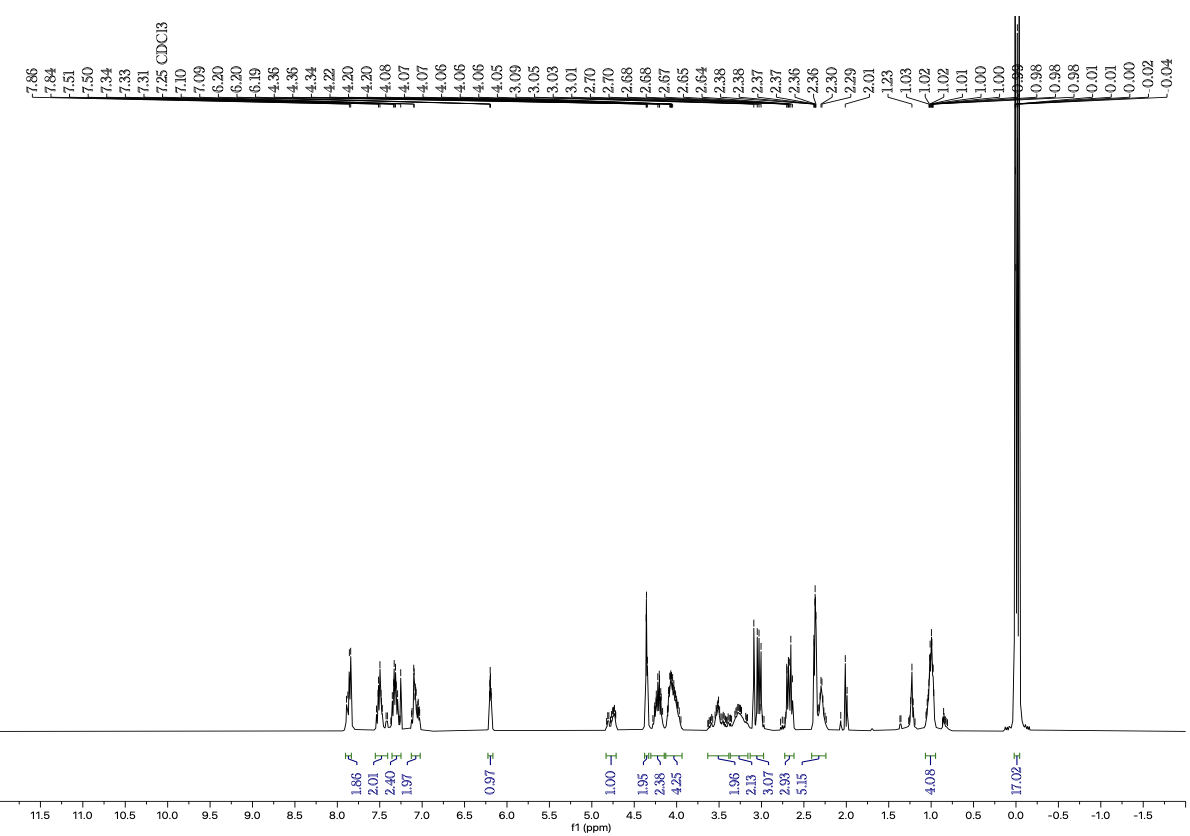


*(^1^H NMR)*


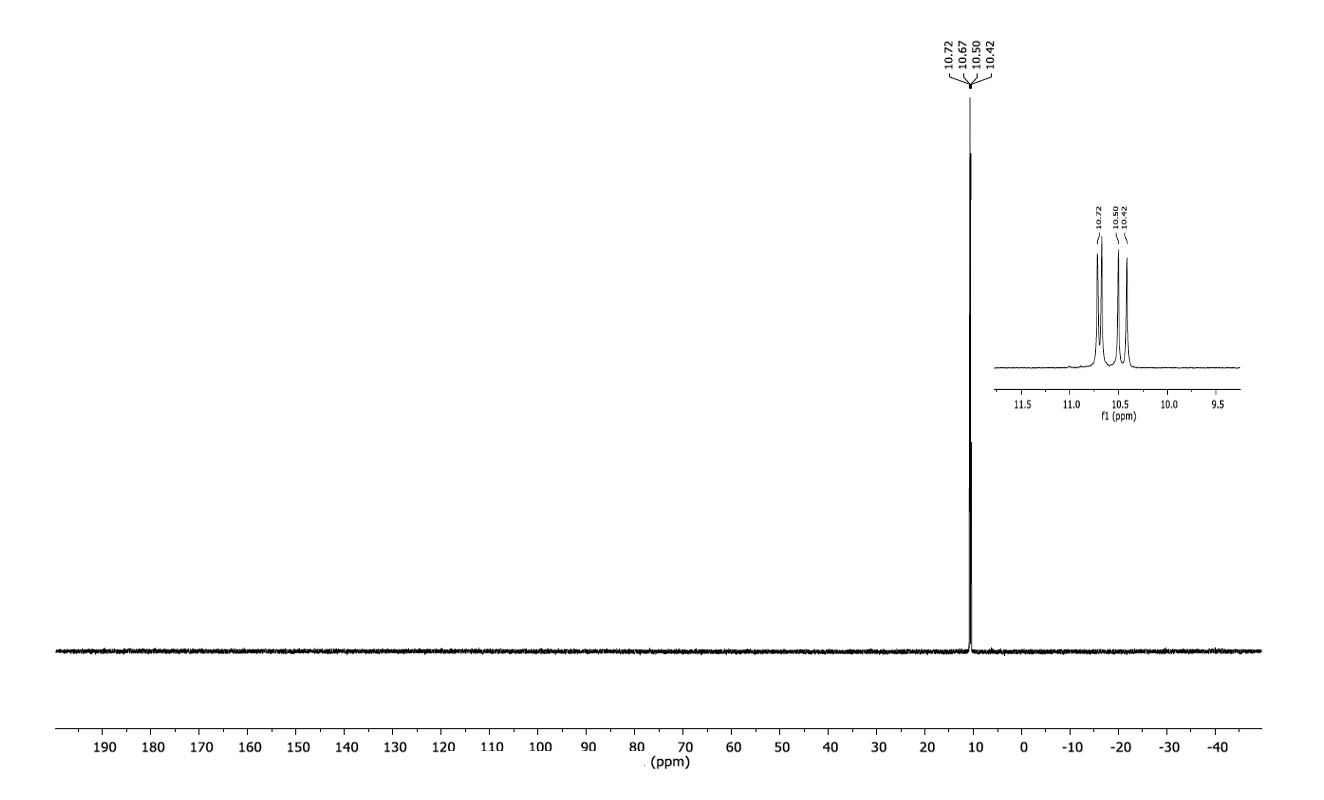


*(^31^P NMR)*


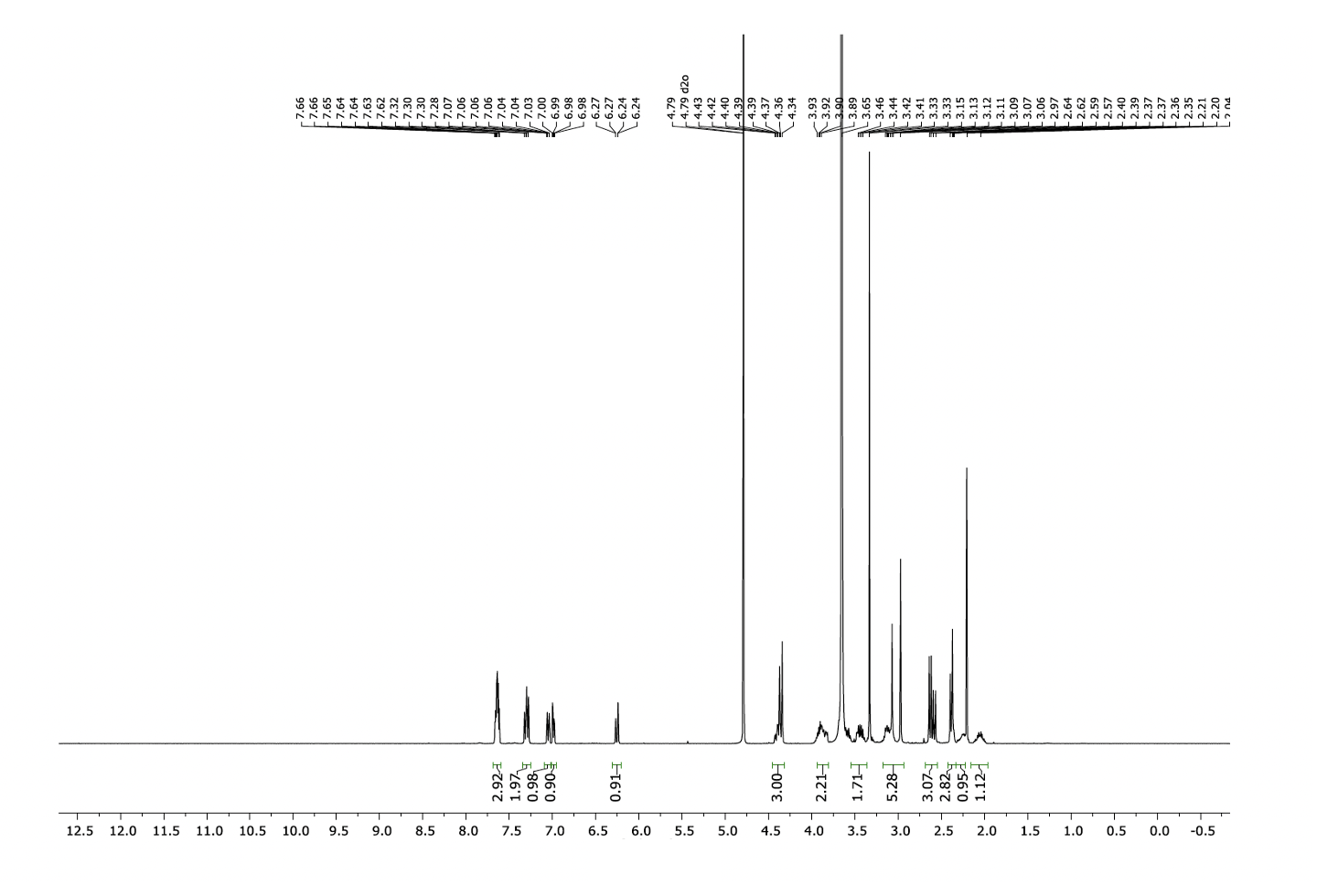


*(^1^H NMR)*


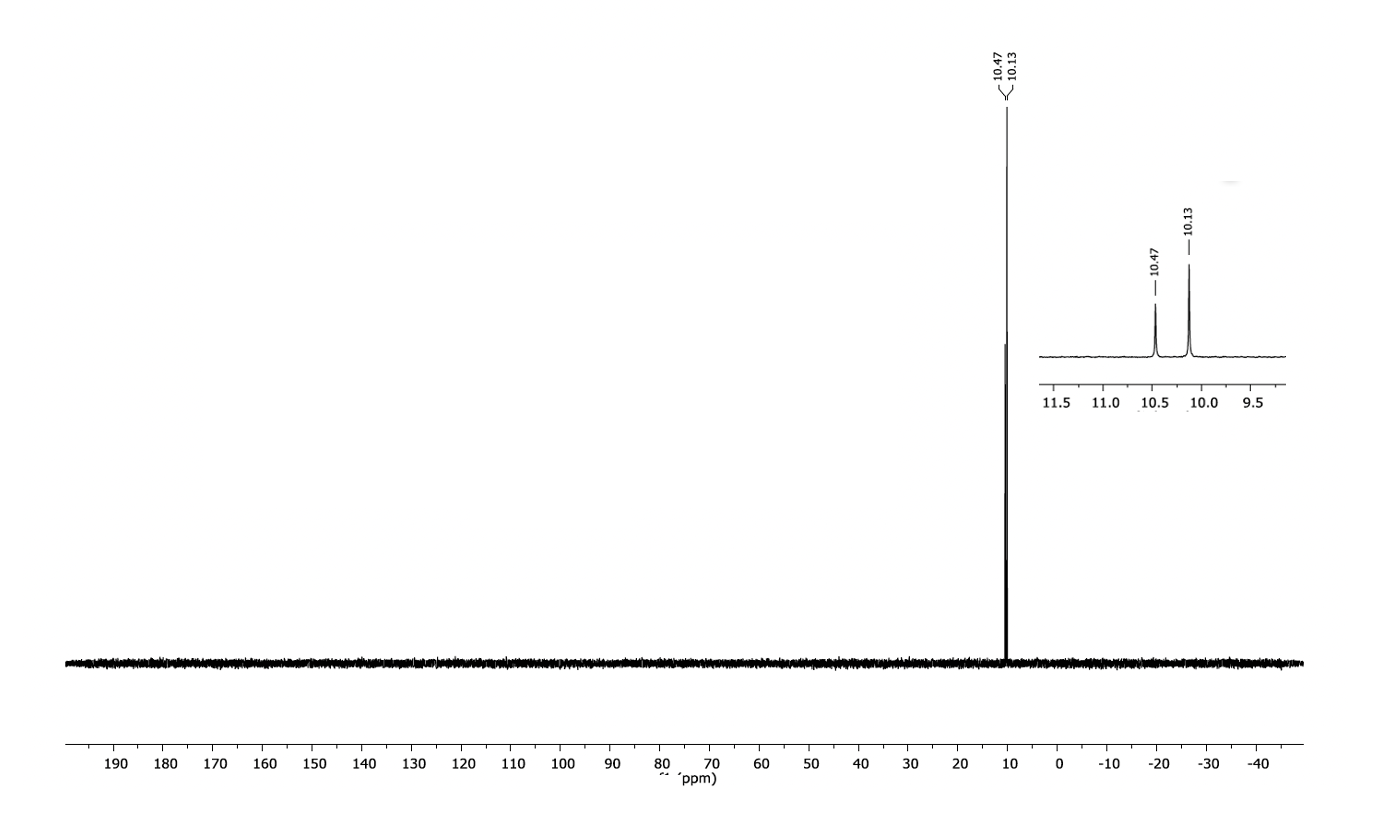


*(^31^P NMR)*


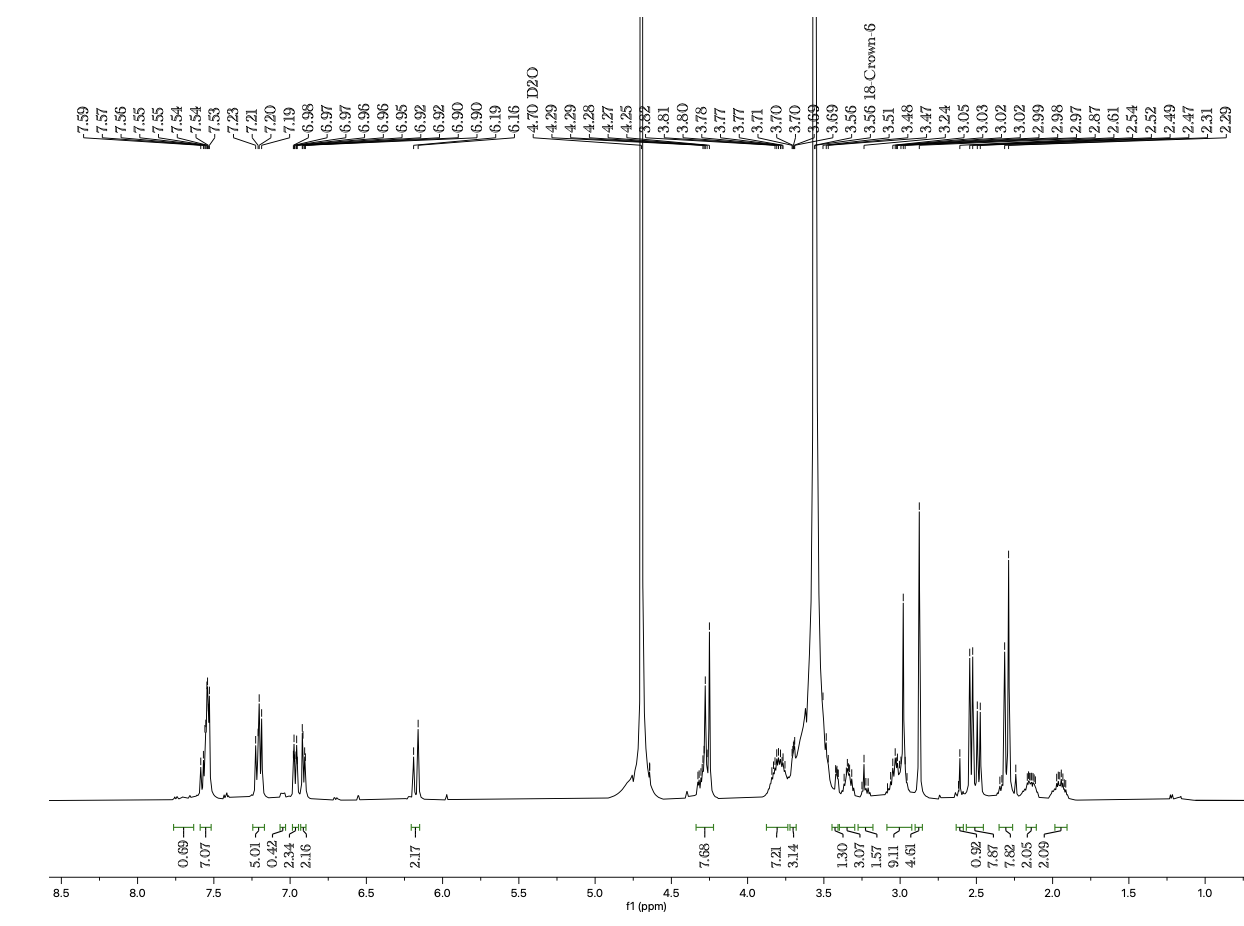


**PSMA-TOP2**

*(^1^H NMR)*


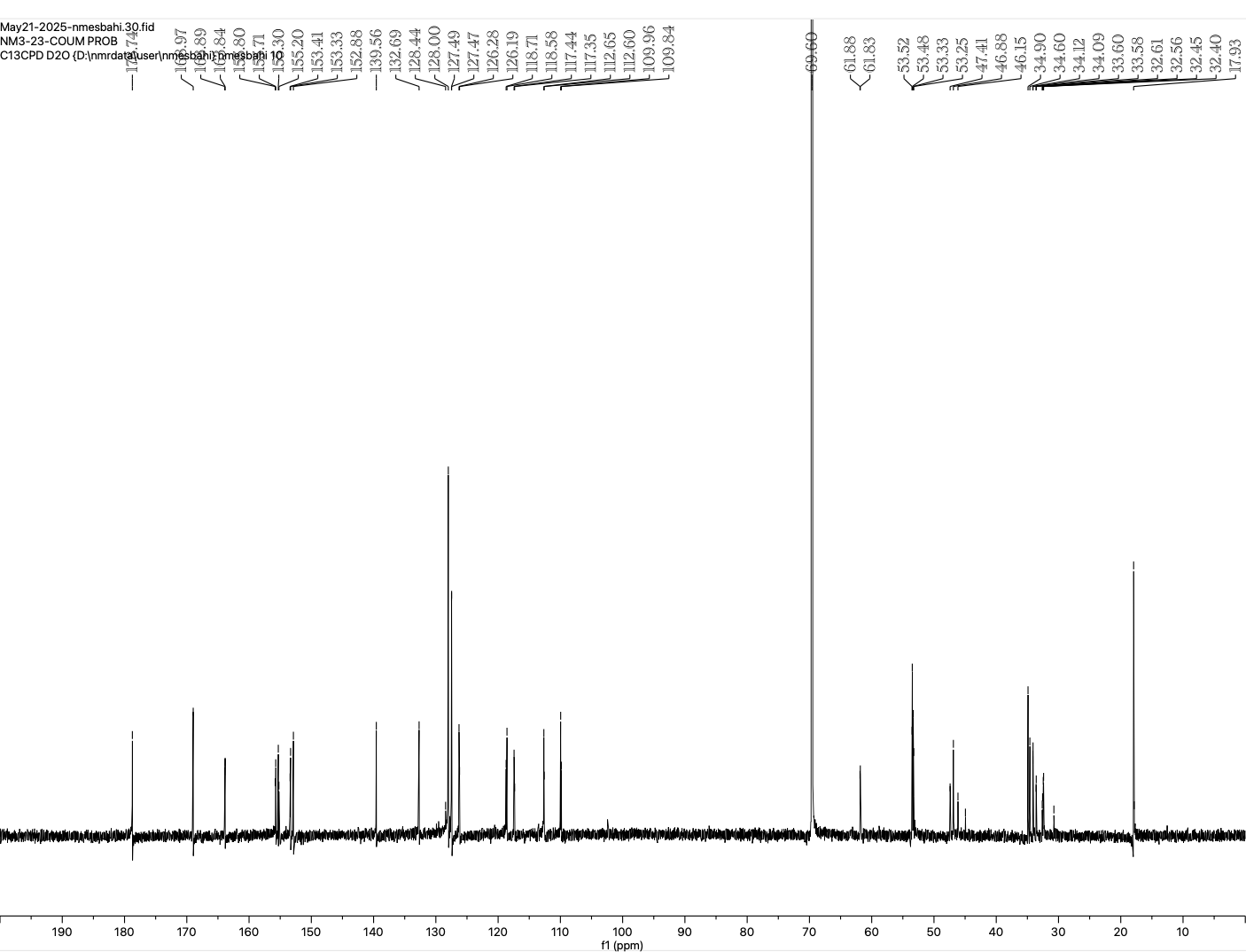


**PSMA-TOP2**

*(^13^C NMR)*


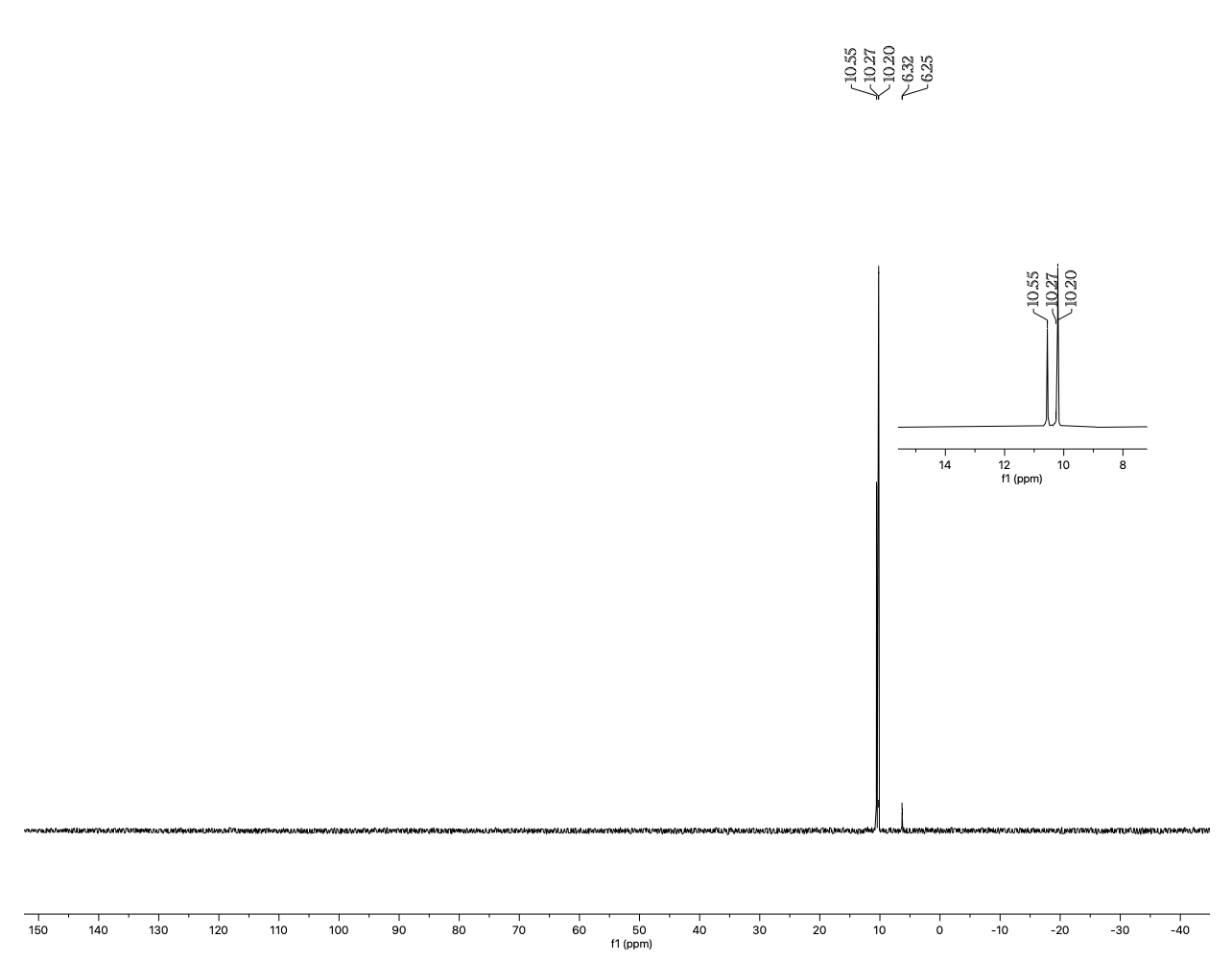


**PSMA-TOP2**

*(^31^P NMR)*

**V. HRMS Spectrum of PSMA-TOP2**

m/z calculated for C_69_H_85_N_11_O_25_P_2_^-^ [M-H]^–^: 1529.52, found 1528.5115.


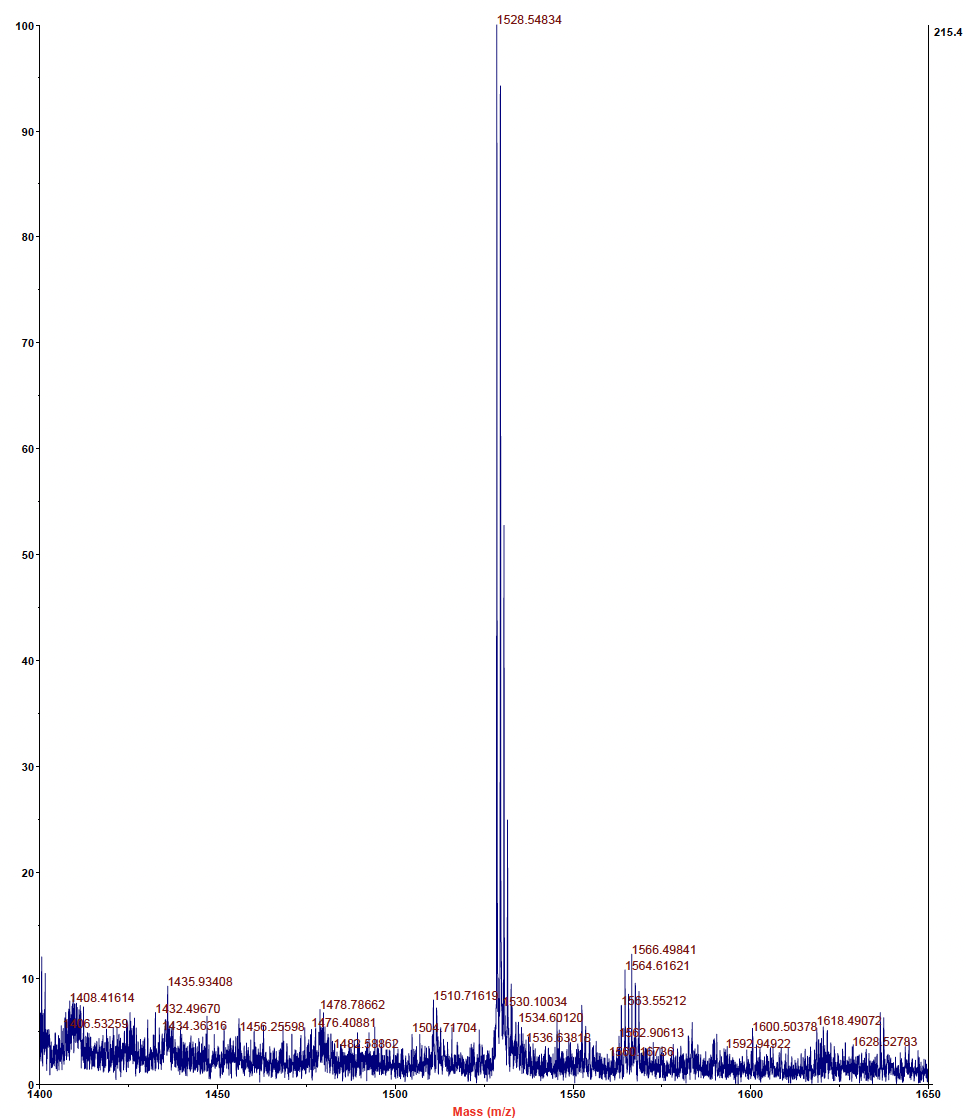


**VI. HPLC Methods and Chromatogram**

- *Column:* Phenomenex Luna 5 μm C18(2) 100 Å
- *Dimension:* 150 x 4.6 mm
- *Gradient Table:*

| **Time (min)** | **% 10 mM NH_4_OAc** | **% Acetonitrile** | **Flow Rate (mL/min)** |
| --- | --- | --- | --- |
| **0.0** | 95 | 5 | 1 |
| **5.0** | 95 | 5 | 1 |
| **15.0** | 5 | 95 | 1 |
| **20.0** | 5 | 95 | 1 |
| **21.0** | 95 | 5 | 1 |


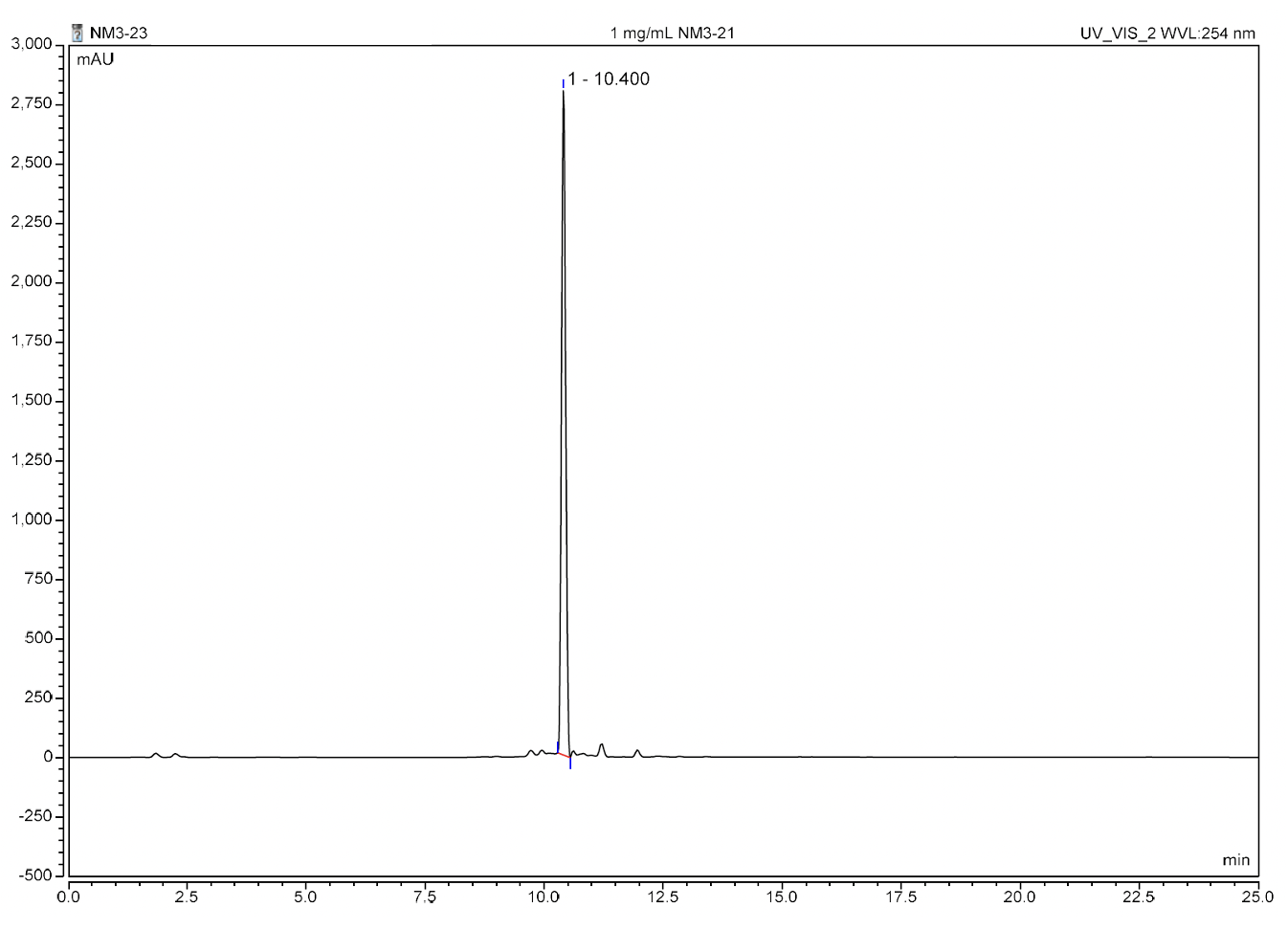


**PSMA-TOP2**

**VII. Inhibitory Potency (IC_50_) of TOP2 to PSMA**

The IC₅₀ value of the TOP2 for PSMA was determined to be 2.0 ± 0.14 nM (SE). The assay for evaluating inhibitory potency toward PSMA was performed as previously described. ^4^ Each assay included three technical replicates for each inhibitor concentration as well as the positive control (absence of inhibitor), and the experiment was repeated at least twice. The IC₅₀ equation [Y = 1/1(1+X/M)], where M represents the calculated IC₅₀, was fitted to each data set using KaleidaGraph version 5.02. Error bars indicate the standard deviation of the samples, while the standard error (SE) is reported with the IC₅₀ value.

**References**

1. Olatunji, F.P., J.W. Herman, B.N. Kesic, D. Olabode, and C.E. Berkman, A click-ready pH-triggered phosphoramidate-based linker for controlled release of monomethyl auristatin E, Tetrahedron Letters, 2020. **61**(41): p. 152398.

2. Yoon, H., E.A. Savoy, N. Mesbahi, et al., A PSMA-targeted doxorubicin small-molecule drug conjugate, Bioorganic & Medicinal Chemistry Letters, 2024. **104**(1): p. 129712.

3. Olatunji, F.P., E.A. Savoy, M. Panteah, N. Mesbahi, A. Abbasi, C.M. Talley, C.L. Lovingier, L.A. Caromile, and C.E. Berkman, Prostate-specific membrane antigen-targeted turn-on probe for imaging cargo release in prostate cancer cells, Bioconjugate Chemistry, 2021. **32**(11): p. 2386–2396.

4. Ley, C.R., N.R. Beattie, S. Dannoon, M. Regan, H. VanBrocklin, and C.E. Berkman, Synthesis and evaluation of constrained phosphoramidate inhibitors of prostate-specific membrane antigen, Bioorganic & Medicinal Chemistry Letters, 2015. **25**(12): p. 2536–2539.
